# League of Mercy

**Published:** 1912-03-23

**Authors:** 


### LEAGUE OF MERCY.

### The South Paddington District.

SPEECHES BY SIR FREDERICK TREVES AND SIR JOSEPH DIMSDALE.

The Annual Meeting of the South Paddington Branch of the League of Mercy was held on March 15 at 19 Hyde Park Terrace, by the courtesy of Mrs. T. Threlfall. The Rev. Prebendary Francis Gurdon, President of the Branch, occupied the chair, and was supported by the lady President, Lady Dimsdale. Mr. Gurdon, in the course of his opening remarks, dwelt on the duty of all sections of the community to share the burden of suffering. He said that none were too poor to help, and if the pennies of the poor could keep up public-houses they might also help to support hospitals. He also spoke gratefully of the gracious telegram sent by the King from India to the annual meeting of the League of Mercy on December 11.

### Sir Frederick Treves' Address.

He said that this was an age of Leagues, but that there was no league of which one could be a member with greater pride than the League of Mercy—the League which helped those who could not help themselves. He pointed out that we who were familiar with our own institutions could not understand how deeply our English system of voluntary hospitals impressed foreigners. It was a spectacle unique in Europe, great hospitals spending hundreds of thousands of pounds a year and maintained entirely by voluntary contributions. The existence of these hospitals showed that the rich felt that it was a privilege to help the poor, and the fact formed a tender bond between class and class. Sir Frederick pointed out, however, that it was not only the poor who benefited by our hospitals. Did we ever pause to think where our doctors came from? Some people seemed to think that they were the product of the State or of the universities, but they were really produced by the hospitals supported by voluntary contributions. If our hospitals were destroyed to-morrow, there would be no means of training medical men. Again, how were new methods of treatment introduced, standardised, and made available? The answer was that these things came through the hospitals. Sir Frederick gave as an instance of this benefit the Finsen light, which cured the terrible disease of progressive lupus. It had cost thousands to introduce the Finsen light. No private medical man could have introduced it. No public body could have done it. It was the hospital that did it, and by gradually developing the method the light had become inexpensive and available for all. The same was true of the *x*-rays, the first installation of which cost a great deal of money, of electricity as used in medicine, and of the treatment of diseases by the injection of various sera. He spoke of Lord Lister, whose work had revolutionised surgery, and made possible and successful operations which before antiseptic treatment was introduced were only dreamed of. It was in the great hospitals where Lister worked that this treatment had been introduced and tested. Some people wished to see hospitals State-supported, but he felt that it would be a lamentable day for England when that came. The rich would lose their great privilege of helping the poor. To municipalise our hospitals would be as if the good Samaritan had been rewarded out of the rates for his kindness to the wounded traveller. Sir Frederick concluded by an allusion to Japan. There one saw many shrines to many gods and goddesses; but the most popular shrine was that of the goddess of mercy. That goddess, he said, was represented with no crown and no wings. But she had a thousand hands.

### Sir Joseph Dimsdale's Speech.

In thanking Sir Frederick Treves for his address, he mentioned that it was forty years since he, as a member of the House Committee of the London Hospital, became interested in the work of a young surgeon named Treves, whose subsequent career had so well fulfilled the expectations of those who had known him in his early days. Sir Joseph recalled also that he had had the privilege of presenting Lord Lister with the honorary freedom of the City of London. He said that nothing would be more deplorable than to hand our hospitals over to the vestries. He thought that too much had been said about the rich in connection with the League of Mercy. The League did not appeal so much to the rich as to the mass of people, those who could give only a shilling—a sixpence—a penny! These little donations showed a spirit of patriotism, and developed in the poor the feeling that they also had done something for the hospitals, and had a status with regard to them. The meeting concluded with a vote of thanks to the hostess, Mrs. Threlfall, for giving the use of her house and for the hospitality she had extended to those present.

